# Allocation of nitrogen and phosphorus in the leaves, stems, and roots of *Artemisia*: a case study in phylogenetic control

**DOI:** 10.3389/fpls.2024.1445831

**Published:** 2024-08-20

**Authors:** Dechun Jiang, Haiyang Gong, Karl J. Niklas, Zhiqiang Wang

**Affiliations:** ^1^ Chengdu Institute of Biology, Chinese Academy of Sciences, Chengdu, China; ^2^ Sichuan Zoige Alpine Wetland Ecosystem National Observation and Research Station, Southwest Minzu University, Chengdu, China; ^3^ College of Grassland Resources, Southwest Minzu University, Chengdu, China; ^4^ School of Integrative Plant Science, Cornell University, Ithaca, NY, United States

**Keywords:** allocation strategy, artemisia, nitrogen, organs, phosphorus, scaling exponent

## Abstract

**Introduction:**

The allocation of nitrogen (N) and phosphorus (P) among plant organs is an important strategy affecting growth and development as well as ecological processes in terrestrial ecosystems. However, due to lack of systematic investigation data, the allocation strategies of N and P in the three primary plant organs (e.g., leaves, stems and roots) are still unclear.

**Methods:**

A total of 912 individuals of 62 *Artemisia* species were examined across a broad environmental expanse in China, and the N and P concentrations of leaves, stems and roots were measured to explore the allocation strategies in different subgenera, ecosystem types, and local sites.

**Results and discussion:**

Across all 62 species, the N vs. P scaling exponents for leaves, stems and roots were 0.67, 0.59 and 0.67, respectively. However, these numerical values differed among subgenera, ecosystem types, and local sites. Overall, the numerical values of N vs. P scaling exponents comply with a 2/3-power function for each *Artemisia* organ-type reflecting a phylogenetically conserved allocation strategy that has nevertheless diversified with respect to local environmental conditions. These results inform our understanding of N and P stoichiometric patterns and responses to abiotic factors in an ecologically broadly distributed angiosperm genus.

## 1 Introduction

The allocation of critical nutrients to different plant organs is an important strategy for growth and development in response to resource limitations and changing environmental conditions that influences the material and energy cycles and ecological processes in terrestrial ecosystems (Bazzaz and Grace, 1997; Sterner and Elser, 2002; Ågren, 2004). Nitrogen (N) and phosphorus (P) are important limiting elements that play crucial roles in plant metabolic pathways, such as photosynthesis, respiration, and reproduction (Elser et al., 2003; 2007; Vitousek, 2004; Sullivan et al., 2014), and contribute significantly to ecosystem functions and biogeochemical cycles (Güsewell, 2004). Leaves, roots and stems are the primary photosynthetic, absorptive, and mechanical organs, respectively, that have different metabolic and structural requirements (Niklas, 2004; Zhao et al., 2016; Lambers and Oliveira, 2019), and therefore likely have different nutrient allocation strategies (Yan et al., 2016; Song et al., 2022; Wang et al., 2022b). Plants need to coordinate the allocation of limiting N and P to maximize their growth and may change allocation strategies in response to different environmental constraints. Thus, exploring the allocation of N and P among the three primary plant organs is of paramount importance for understanding nutrient utilization strategies and predicting ecosystem nutrient fluxes under changing environmental conditions.

Previous studies have explored the N and P allocation strategies in specific organs and tissue systems, such as leaves (Wright et al., 2004; Han et al., 2005; Niklas and Cobb, 2005; Reich et al., 2010; Tian et al., 2018), stems (Wang et al., 2018; Zhang et al., 2018; 2022a; Zhao et al., 2021), fine roots (Yuan et al., 2011; Wang et al., 2019, 2021), seeds (Wang et al., 2020), and bark (Gong et al., 2024). Using comprehensive global data sets, two empirical studies have asserted that specific stoichiometric allocation patterns hold true for leaves (Reich et al., 2010) and fine roots (Wang et al., 2019). However, other studies have reported that the numerical values of the N vs. P scaling exponent differ in response to ambient environmental factors and across different lineages (Kerkhoff et al., 2006; Niklas, 2006; Tian et al., 2018; Wang et al., 2021).

Arguably, despite considerable progress, our understanding of plant N and P allocation strategies remains limited for at least two important reasons. First, many previous studies are based on data compilations drawn from the primary literature, which conflates different survey approaches and methods. And second, few studies have explored the effects of phylogeny on N and P allocation patterns, which may differ across different lineages and clades. In an effort to compensate for these potential difficulties, this study focused on a single genus, *Artemisia*, which includes more than 500 herbaceous species in a species-rich and ecologically important family (Malik et al., 2017). In addition to its architectural diversity, *Artemisia* was selected because of its wide distribution in different habitats and climate zones across Asia, Europe, and North America (Liu et al., 2021; Liu et al., 2023). A third benefit is that the genus has several closely related late divergent subgenera (Yang et al., 2015) that appear to share similar trait-evolution trajectories making the genus an ideal resource with which to explore N and P allocation strategies. Consequently, The N and P concentrations for leaves, stems and roots of 912 individuals of 62 *Artemisia* species were measured along a broad environmentally diverse expanse in China to determine N and P allocation strategies among the three primary plant organs.

The goals of this study were to determine (1) whether there are differences in leaf, stem, and root N and P concentrations and N:P ratios among subgenera and ecosystem types, (2) whether the numerical values of N vs. P scaling exponents observed for different organs, subgenera, and ecosystem types conform to previous asserted power-law “rules”, and (3) whether the numerical values of N vs. P scaling exponents of the three major organs differ predictably across local sites, and climatic and soil conditions?

## 2 Materials and methods

### 2.1 Field sampling and measurement

**Figure 1:**
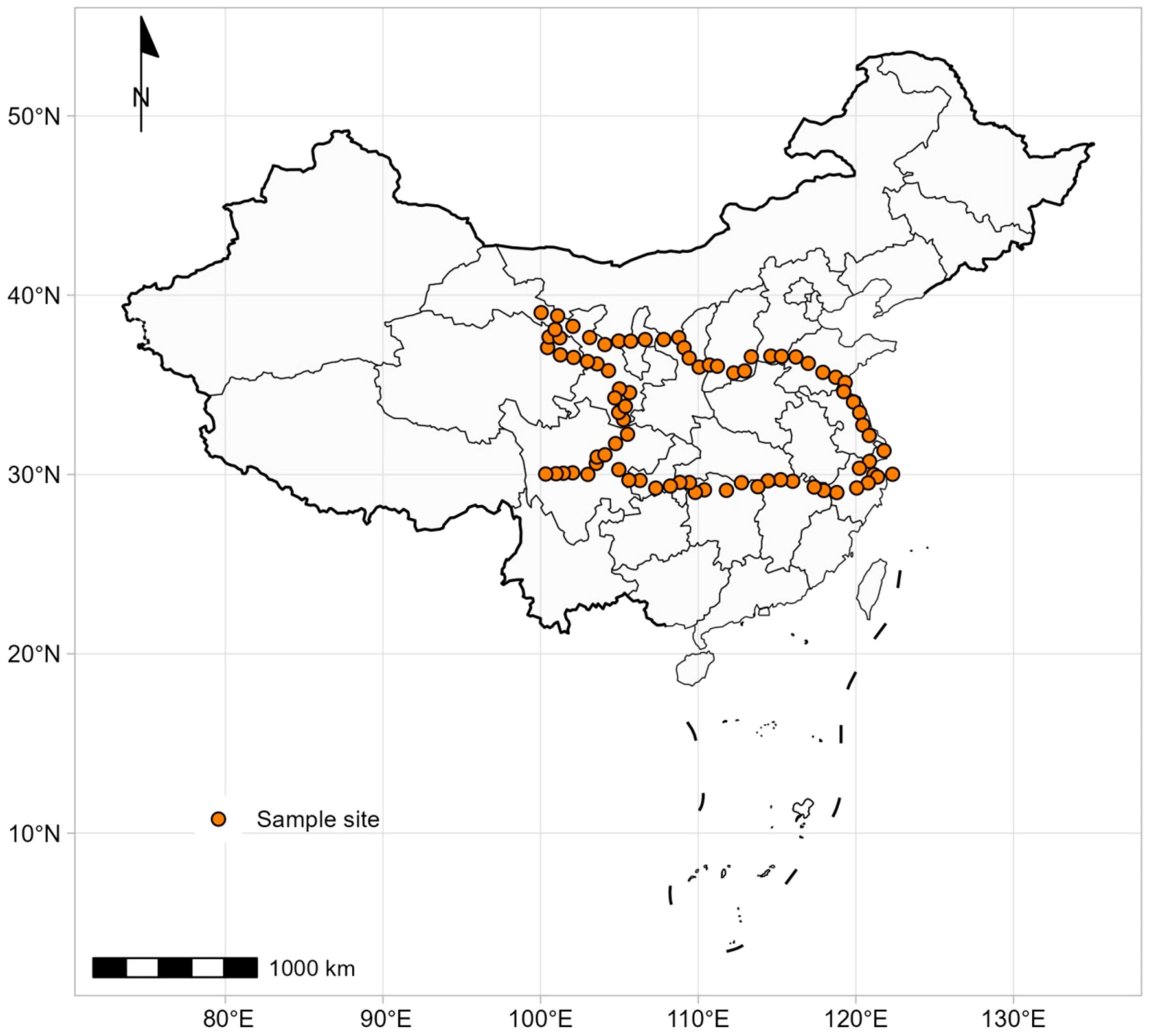
The distribution of the 81 sampling sites used in this study.

These data were collected from a previous study, and the detail sampling information also see Liu et al. (2023). A total of 912 individuals of 62 *Artemisia* species were sampled from 81 sites across China between late July and August 2018 (**Figure 1**). The majority of plants (> 95%) were at the flowering stage. Among the 62 *Artemisia* species, eight were in the subgenera *Absinthium*, 18 in the subgenera *Dracunculus*, and 36 in the subgenera *Artemisia*. The sampling sites covered four ecosystem types (desert, shrubland, grassland, and forest).

Individual plants were divided into leaves, stems and roots, which were dried at 65°C for 48h to obtain dry mass. The organs were then ground to fine powder and passed through a 100-mesh sieve. The N concentration was measured using an elemental analyzer (Vario EL III, Elementar, Hanau, Germany), and the P concentration was determined using a Thermo Scientific iCAP 6300 (Thermo Fisher Scientific Inc., Waltham, MA, USA) (also see Liu et al., 2023). Leaf dry matter content (LDMC), stem dry matter content (SDMC), and root dry matter content (RDMC) were calculated as the quotient of dry mass to fresh mass.

### 2.2 Statistical analysis

Here, we take a scaling approach to evaluate the allocation of N and P among plant organs, which can be described by a power function in the form of N = *β*P*^α^*, which when log-transformed takes the form logN = log*β* + *α*logP, where *α* and *β* are the log-log regression slope (the scaling exponent) and the regression intercept (the normalization constant), respectively (Niklas, 1994, 2006; Kerkhoff et al., 2006; Ågren, 2008). In the context of N and P allocation, the numerical value of the scaling exponent (*α*) reflects the trade-off between N and P investment (Wright et al., 2005; Elser et al., 2010). Numerical values equal to unity indicate an equitable (isometric) N vs. P allocation, whereas values unequal to unity indicate a biased (allometric) allocation, as for example predicted by the growth rate hypothesis, which predicts α < 1 (Elser et al., 2000, 2003; Sterner and Elser, 2002; Yang et al., 2014).

One-way ANOVA with Student’s test was used to compare the differences in the N and P concentrations and N:P ratios of leaves, stems and roots among different subgenera and ecosystem types. N and P concentrations were log_10_-transformed to assure normality. Standard major axis (SMA) in the lmodel2 function of the R package LMODEL2 was used to determine N vs. P scaling exponents (Legendre, 2018). The likelihood ratio test was used to examine the heterogeneity of N vs. P scaling exponents among the subgenera and different ecosystem types. Given that tissue dry matter content is negatively correlated with photosynthetic capacity (Santiago et al., 2004), tissue dry matter content was used as a reasonable surrogate for growth rates (Niklas, 2004; Chave et al., 2009). SMA regression was used to evaluate the scaling relationships of N and P concentrations with respect to LDMC, SDMC and RDMC. To determine the numerical values of the N vs. P scaling exponents of different organs at each site, species with 10 or more samples from each site were selected (a total of 21 sites for leaves, 16 sites for stems, and 21 sites for roots) for SMA regression analyses. All the data analyses were performed in R 4.3.1 (R Development Core Team, 2023).

## 3 Results

### 3.1 Leaf, stem, and root N and P concentrations and N:P ratios in different subgenera and ecosystem types

Across all observations, the average N concentrations were 24.25, 7.26 and 7.53 mg/g in the leaves, stems and roots, respectively. The average P concentrations were 2.07, 1.17 and 1.04 mg/g, respectively. The average N:P ratios were 11.74, 6.19 and 7.23, respectively (**Table 1**). The N and P concentrations and N:P ratios differed significantly among different subgenera and ecosystem types (**Table 1**). The subgenera *Absinthium* had the highest N concentration and N:P ratio and the lowest P concentration of all organs, whereas the subgenera *Artemisia* had the lowest N concentration and N:P ratio and the highest P concentration of all organs. Among the different ecosystem types, deserts had the highest N concentrations and N:P ratios and the lowest P concentrations of all organs. Forests had the lowest N concentrations and N:P ratios, whereas grassland had the highest P concentrations.

**Table 1:** Nitrogen (N) and phosphorus (P) and N:P ratios among different organ of the 62 *Artemisia* species in different subgenera and ecosystems.

	*n*	Leaf N (mg/g)	Leaf P (mg/g)	Leaf N:P	Stem N (mg/g)	Stem P (mg/g)	Stem N:P	Root N (mg/g)	Root P (mg/g)	Root N:P
All	912	24.25 ± 0.21	2.07 ± 0.03	11.74 ± 0.17	7.26 ± 0.09	1.17 ± 0.02	6.19 ± 0.16	7.53 ± 0.09	1.04 ± 0.18	7.23 ± 0.14
Subgenera										
Absinthium	70	26.81 ± 0.95a	1.92 ± 0.11a	13.99 ± 0.90a	9.74 ± 0.32a	1.01 ± 0.06b	9.62 ± 0.66a	8.95 ± 0.24a	0.90 ± 0.05b	9.92 ± 0.50a
Artemisia	583	23.85 ± 0.40b	2.08 ± 0.03a	11.53 ± 0.38b	7.01 ± 0.10b	1.26 ± 0.03a	5.56 ± 0.18c	7.32 ± 0.11b	1.10 ± 0.02a	6.68 ± 0.16c
Dracunculus	259	24.13 ± 0.25b	2.07 ± 0.03a	11.59 ± 0.18b	7.24 ± 0.16b	1.03 ± 0.04b	7.01 ± 0.32b	7.65 ± 0.18b	0.96 ± 0.03b	7.95 ± 0.27b
Ecosystem type										
Desert	92	27.73 ± 0.73a	1.63 ± 0.08b	17.02 ± 0.68a	10.14 ± 0.29a	0.80 ± 0.05c	12.71 ± 0.64a	9.27 ± 0.37a	0.80 ± 0.06b	11.55 ± 0.52a
Shrubland	143	24.43 ± 0.56b	1.77 ± 0.07b	13.79 ± 0.51b	7.63 ± 0.21b	0.91 ± 0.05b	8.38 ± 0.40b	8.29 ± 0.25b	0.82 ± 0.05b	10.12 ± 0.39b
Grassland	169	23.98 ± 0.44b	2.23 ± 0.05a	10.81 ± 0.17c	7.42 ± 0.23b	1.30 ± 0.04a	5.73 ± 0.34c	7.65 ± 0.23b	1.17 ± 0.04a	6.52 ± 0.26c
Forest	508	23.70 ± 0.27b	2.19 ± 0.04a	10.73 ± 0.34c	6.68 ± 0.09c	1.30 ± 0.03a	5.13 ± 0.14c	7.01 ± 0.10c	1.12 ± 0.02a	6.26 ± 0.13c

n is the number of samples. Different letters indicate significant differences at 0.05 level.

### 3.2 Leaf, stem, and root N versus P scaling exponents across subgenera, ecosystem types, and local sites

**Figure 2:**
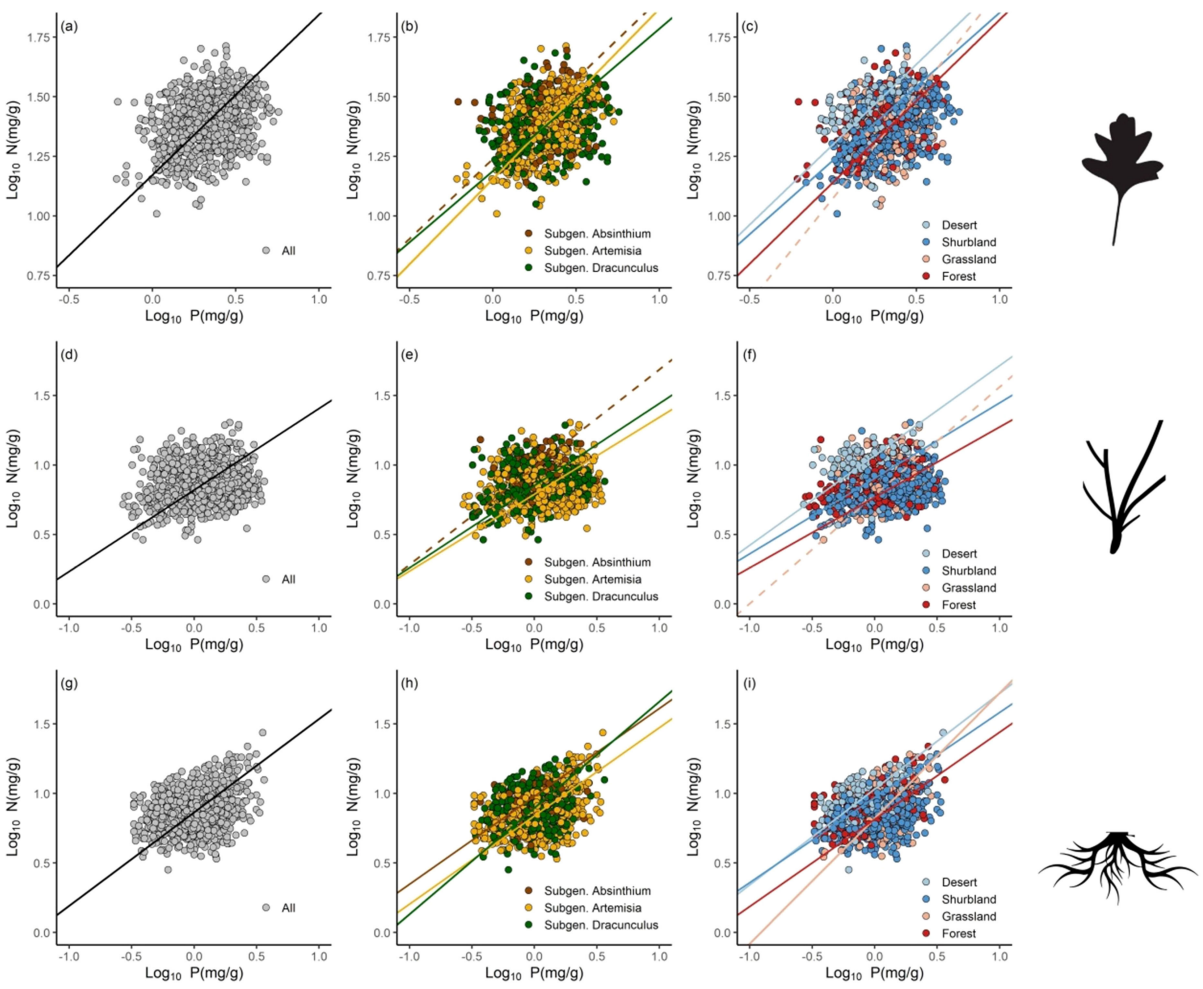
Scaling relationships of nitrogen (N) and phosphorus (P) for leaves **(A-C)**, stems **(D-F)** and roots **(G-I)** in pooled data, and different subgenera and ecosystem types.

Across all observations, the N vs. P scaling exponents were 0.67 (*r* ^2^ = 0.08, *P*< 0.001), 0.59 (*r* ^2^ = 0.02, *P*< 0.001), and 0.67 (*r* ^2^ = 0.09, *P*< 0.001) for leaves, stem and roots, respectively (**Figure 2**; **Table 2**). Among the subgenera, there were significant differences in the numerical values of exponents. For example, the subgenera *Artemisia* had higher leaf N vs. P scaling exponent (*α* = 0.71) than the subgenera *Dracunculus* (*α* = 0.60). In contrast, the *Dracunculus* had the highest root N vs. P scaling exponent (*α* = 0.77) than the subgenera *Artemisia* (*α* = 0.64) and the *Absinthium* (*α* = 0.63). Likewise, significant differences in the N vs. P scaling exponents were found across ecosystem types. In the case of leaves, forests had the highest N vs. P scaling exponent (*α* = 0.68) compared to deserts (*α* = 0.67) and shrublands (*α* = 0.62). The stem N vs. P scaling exponent for deserts was 0.65, whereas that of shrublands and forests were 0.54 and 0.51, respectively. The root N vs. P scaling exponent for grasslands was 0.90, whereas that of shrublands, deserts, and forests were 0.61, 0.69 and 0.63, respectively.

**Table 2:** Summary of standard major axis (SMA) regression results between N and P concentrations among different organs of the 62 *Artemisia* species in different subgenera and ecosystems.

		Leaf				Stem				Root			
Plant group	*n*	Intercept	Exponent (95% CIs)	*r* ^2^	*P*	Intercept	Exponent (95% CIs)	*r* ^2^	*P*	Intercept	Exponent (95% CIs)	*r* ^2^	*P*
All	912	1.17	0.67(0.63-0.72)	0.08	< 0.001	0.82	0.59(0.55-0.63)	0.02	< 0.001	0.87	0.67(0.63-0.72)	0.09	< 0.001
Subgenera													
Absinthium	70	——	——	——	0.11	——	——	——	0.3	0.98	0.63(0.50-0.80)b	0.06	0.049
Artemisia	583	1.15	0.71(0.66-0.77)a	0.17	< 0.001	0.79	0.55(0.51-0.60)a	0.05	< 0.001	0.84	0.64(0.59-0.68)b	0.14	< 0.001
Dracunculus	259	1.19	0.60(0.53-0.68)b	0.02	0.045	0.85	0.59(0.52-0.67)a	0.02	0.01	0.9	0.77(0.68-0.86)a	0.05	< 0.001
Ecosystem type													
Desert	92	1.3	0.67(0.55-0.82)a	0.07	0.012	1.07	0.65(0.53-0.79)a	0.12	< 0.001	1.03	0.69(0.58-0.82)b	0.29	< 0.001
Shrubland	143	1.23	0.62(0.53-0.72)a	0.16	< 0.001	0.9	0.54(0.47-0.63)b	0.16	< 0.001	0.97	0.61(0.53-0.70)b	0.30	< 0.001
Grassland	169	——	——	——	0.574	——	——	——	0.172	0.82	0.90(0.78-1.04)a	0.06	< 0.001
Forest	508	1.14	0.68(0.63-0.74)a	0.18	< 0.001	0.77	0.51(0.47-0.55)b	0.13	< 0.001	0.82	0.63(0.58-0.68)b	0.15	< 0.001

n is the number of samples. Different letters indicate significant differences at 0.05 level.

**Figure 3:**
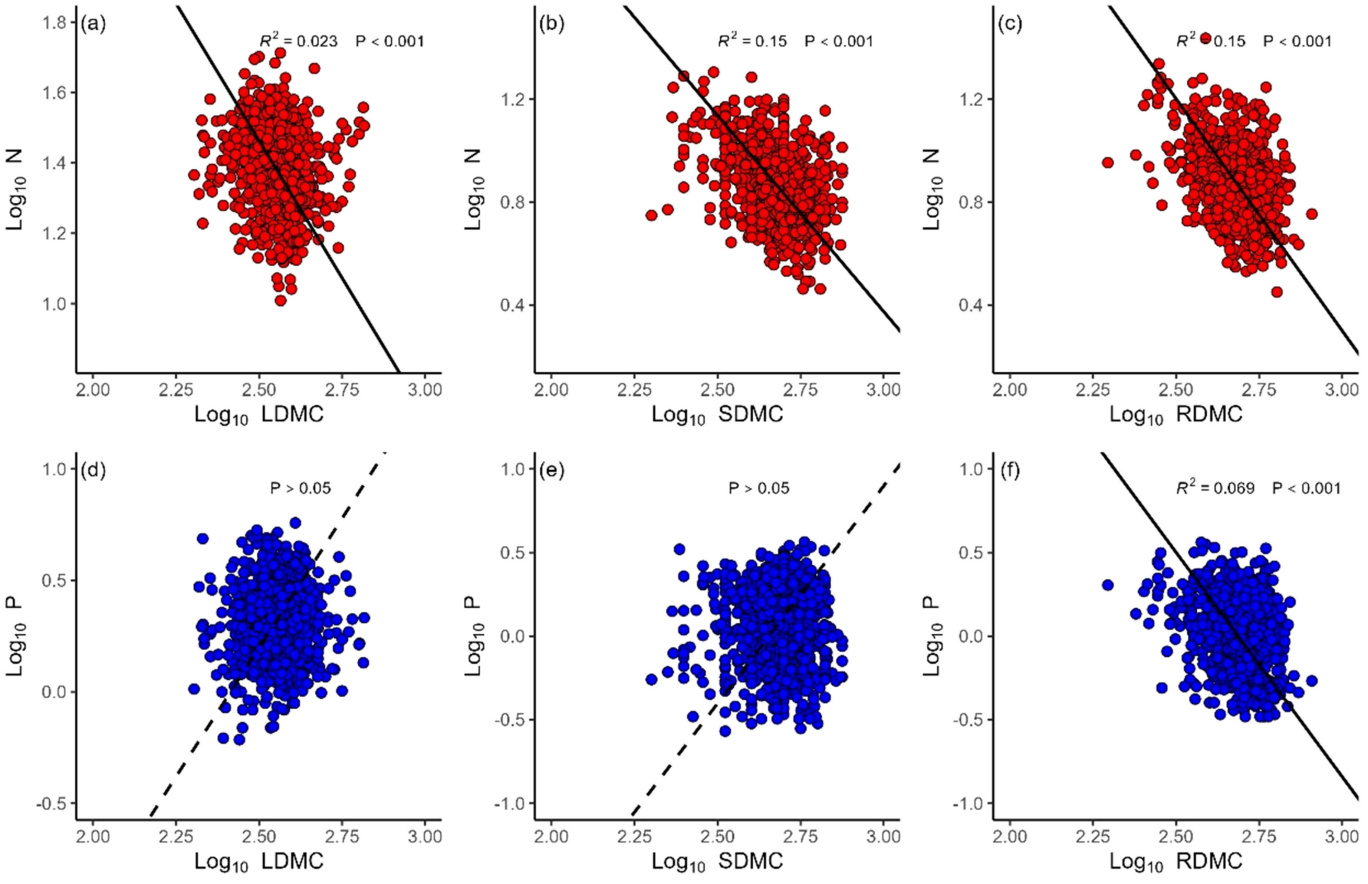
The relationships of leaf (LDMC), stem (SDMC) and root (RDMC) dry matter content and nitrogen (N) **(A-C)**, and phosphorus (P) **(D-F)**.

**Figure 4:**
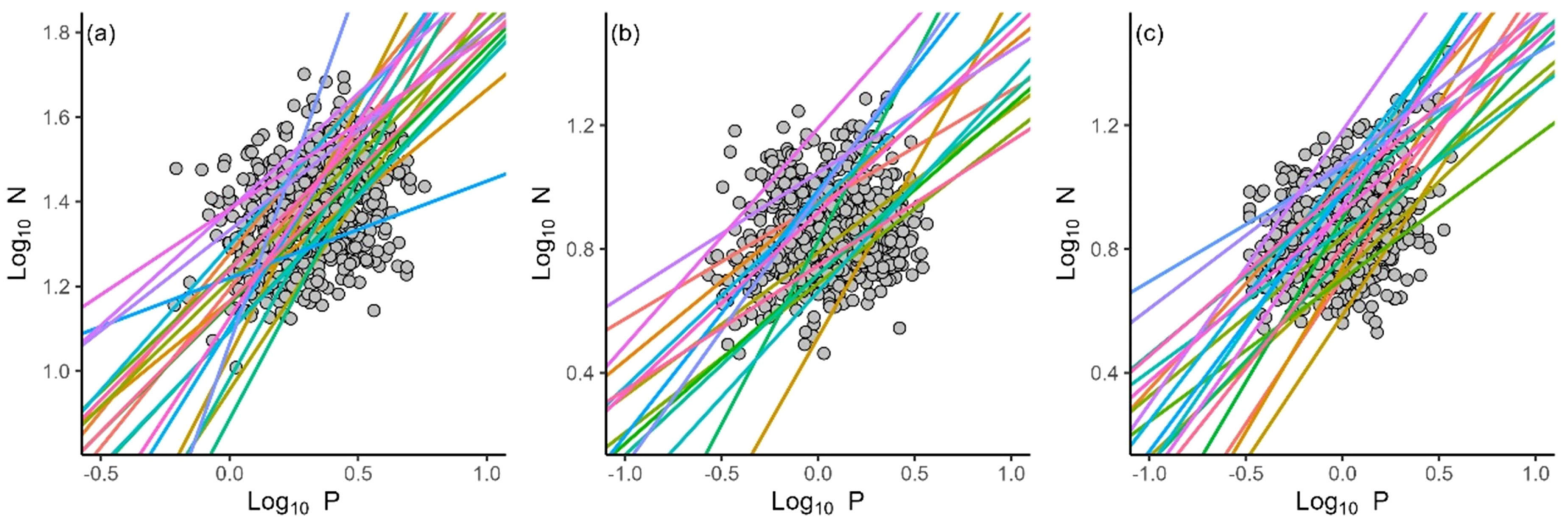
The N and P scaling relationships of leaves **(A)**, stems **(B)** and roots **(C)** for different local sites.

N concentrations decreased with LDMC (*α* = –1.56, *r* ^2^ = 0.02, *P*< 0.001), SDMC (*α* = –1.52, *r* ^2^ = 0.15, *P* < 0.001) and RDMC (*α* = –1.80, *r* ^2^ = 0.15, *P* < 0.001) (**Figures 3A-C**). P concentrations decreased with RDMC (*α* = –2.68, *r* ^2^ = 0.07, *P* < 0.001), whereas P concentrations did not show significant relationships with either LDMC or SDNC (*P* > 0.05) (**Figure 3D-F**). The N vs. P scaling exponents of all organs statistically differed among local sites (**Figure 4**). The leaf N vs. P scaling exponent ranged from 0.23 to 1.70 with a mean of 0.75. The stem N vs. P scaling exponent ranged from 0.37 to 1.18 with a mean of 0.64, whereas the root N vs. P scaling exponent ranged from 0.37 to 1.06 with a mean of 0.71.

## 4 Discussion

### 4.1 N and P concentrations and N:P ratio in leaves, stems and roots of Artemisia species

The results show that N and P concentrations and N:P ratios differ significantly among the three plant organs of *Artemisia* species (**Table 1**). Leaves have higher N and P concentrations and N:P ratios compared to stems and roots, which accords with previous studies (Kerkhoff et al., 2006; Zhang et al., 2018; Chen et al., 2020; Zhao et al., 2021) pointing to the fact that leaves require more N and P to maximize the energy requirements for their physiological activities (Wright et al., 2005; Zhao et al., 2019; Zhao et al., 2021). As noted by others, leaves are rich in Rubisco, which contains significant amounts of N (Ghimire et al., 2017) that is positively associated with high photosynthetic and respiratory rates (Wright et al., 2004; Donovan et al., 2011). In contrast, stems and roots share similar N and P concentrations and N:P ratios likely because they generally have lower metabolic rates and contain larger amounts of storage and mechanically supportive tissues (rich in cellulose, hemicellulose, and lignin) requiring lower N and P concentrations for their metabolic activities (Kerkhoff et al., 2006; Zhang et al., 2018).

Despite the relatively small phylogenetic distances among the three *Artemisia* subgenera, significant differences in N and P concentrations as well as N:P ratio are seen in leaves, stems and roots (**Table 1**). For example, compared to the other two subgenera, *Absinthium* had higher N concentrations and N:P ratios but lower P concentrations, indicating that phylogenetic legacy cannot be discounted when assessing interspecific trends in allocation patterns (Kerkhoff et al., 2006; Paoli, 2006; Townsend et al., 2007). Hao et al. (2015) report that the factors “genus” and “species” on average account for 59% of the total variation in N and P concentrations within the subfamily Didymocarpoideae in China.

In addition, *Artemisia* N and P concentrations and N:P ratios differ significantly across different ecosystem types (**Table 1**). For example, the N concentrations and N:P ratios of *Artemisia* species are higher in deserts than in shrublands, grasslands, or forests, whereas N concentrations and N:P ratios are lower in forests than in grasslands, shrublands, or deserts. In contrast, *Artemisia* species growing in forests have lower N concentrations and N:P ratios as a result of differences in species composition (Yang et al., 2015). These data support the idea that species adapted to dry conditions tend to invest more N in the construction of their organs, perhaps to maintain growth by reducing stomatal conductance and increasing water use efficiency (Cunningham et al., 1999; Wright et al., 2003; Sardans and Peñuelas, 2013).

### 4.2 N vs. P scaling exponents in leaves, stems and roots of Artemisia species

The data reveal that the numerical values of N vs. P scaling exponents are all less than unity for each of the organ types of all *Artemisia* species (**Figure 2**; **Table 2**), indicating a disproportionate investment in P compared to N. These results are statistically indistinguishable from a 2/3-power law reported by Reich et al. (2010) using a global dataset and support the growth rate hypothesis. In contrast, the N vs. P scaling exponent for roots (i.e., α = 0.67) is inconsistent with the 0.82-power law reported by Wang et al. (2019). Perhaps more interesting, the stem N vs. P scaling exponent (i.e., α = 0.59) is numerically close to 2/3, which is observed for the leaves and roots examined in this study. This observation likely reflects the fact that all species examined in our study are herbaceous and typically possess photosynthetic green stems (Yang et al., 2016), which can contribute to overall growth (Adler et al., 2014), but require a greater supply of N and P. Therefore, it is not surprising that the stem N vs. P scaling exponent is similar to leaves.

We speculate that the scaling exponents of leaves, stems, and roots are numerically convergent as a result of the close phyletic history shared by the species in each of the three subgenera (McGroddy et al., 2004). *Artemisia* species all share a long and similar evolutionary history, which may confine their allocation strategies to a comparatively narrow range of nutrient requirements (see Cavender-Bares et al., 2020). Previous studies similarly reported “invariant” N vs. P scaling exponents for leaves, stems and roots among herbaceous and woody species along different environmental gradients the Changbai Mountain of China (Zhao et al., 2016), a north-south transect of eastern China (Zhang et al., 2018) that, along with our study, provide strong evidence that N and P scaling relationships can be highly conserved among related species (Kerkhoff et al., 2006; Chen et al., 2020).

Leaf, stem, and root dry matter content is known to be an important functional plant trait (Cornelissen et al., 2003) and previous studies have shown that tissue dry matter content is negatively correlated with photosynthetic capacity (Santiago et al., 2004), which can be a surrogate measurement for plant growth rates (Chave et al., 2009). Therefore, we evaluated whether the growth rate hypothesis of P and N requirements was consistent with the scaling exponents for P and N. The results indicate that the scaling of root P vs. RDMC has a numerically larger scaling exponent than the scaling exponent of root N vs. RDMC (**Figure 3**). This observation is consistent with the growth rate hypothesis, which predicts that plants with greater metabolic rates require more P than N to support their elevated protein synthesis demands. It is particularly surprising therefore that leaf and stem P manifest no significant relationship with LDMC and SDMC (*P >*0.05). It is possible that herbivores and pathogens affected our sampling of above-ground biomass (even though there was no obvious damage to the leaves and stems collected in this study). Consequently, future work is warranted to examine the relationship of stem and leaf N and P contents with LDMC and SDMC.

### 4.3 Variations of N vs. P scaling exponents across ecosystem types and local sites

The data reveal statistically significant differences in N vs. P scaling exponents with respect to ecosystem types and local sites (**Figures 3**, **4**; **Table 2**), indicating differences N and P allocation strategies in organs in response to local environmental conditions. We speculate that regional environmental factors, such as climate, soil nutrient availability, and even species composition, play important roles in determining N vs. P scaling exponents for each of the three primary plant organs, even within this otherwise tight-nit genus. Many previous studies have shown that plants are capable of modifying metabolic rates and may change allocation strategies to adapt to local climate and soil nutrients constraints (e.g., Craine et al., 2005; Liu et al., 2010; Guo et al., 2021). For example, desert plant species generally allocate more N in stems compared to species adapted to less arid conditions to maintain physiological activity, particularly to more effectively transport photosynthates and water to roots (Marschner et al., 1997), and to provide mechanical support (Niklas, 1992; West et al., 1999). Likewise, species growing in shrublands tend to accumulate P more rapidly in roots, perhaps to compete for limited nutrients by means of mycorrhizal symbiotic associations (Lambers et al., 2008; Onipchenko et al., 2009; Yan et al., 2023). Hence, our findings regarding differences in N and P allocation strategies are not surprising except that they are evidence even within a phylogenetically well-defined and otherwise fairly uniform species complex. However, it must be noted that due to the limited latitude range in our study, we cannot evaluate the effects of climatic and soil physicochemical factors on the N vs. P scaling exponents at local sites. Our understanding of the nutrients allocation strategies in local sites is still severely restricted. Future work on a larger scale is required and necessitates further investigation.

## 5 Conclusions

Based on 62 *Artemisia* species growing within an ecologically diverse and wide expanse in China, the N vs. P scaling exponents for leaves, stems and roots are 0.67, 0.59 and 0.67, respectively, which is consistent with a 2/3–power rule thereby supporting the growth rate hypothesis. These results confirm that N vs. P scaling exponents are highly conserved for the three primary plant organs within a phylogenetically well-defined species-complex, although these scaling exponents differ numerically across the three subgenera, ecosystem types, and local sites. These results help to better clarify our current understanding of plant nutrient allocation strategies by confining the results of analyses to a phyletically well-defined group using the same sampling and measurement protocols.

## Data Availability

The original contributions presented in the study are included in the article/supplementary material. Further inquiries can be directed to the corresponding author.
